# Quality Analysis of Long dan Xie gan Pill by a Combination of Fingerprint and Multicomponent Quantification with Chemometrics Analysis

**DOI:** 10.1155/2018/4105092

**Published:** 2018-12-11

**Authors:** Jing Liu, Hui Liu, Zhong Dai, Shuangcheng Ma

**Affiliations:** ^1^National Institutes for Food and Drug Control, Beijing 100050, China; ^2^Institute of Food and Drug, Yanbian Korean Autonomous Prefecture, Jilin Province 133002, China

## Abstract

Long dan Xie gan pill is a traditional complex compound preparation with a long history for treatment of diseases, including hepatocolic hygropyrexia, dizziness, tinnitus, and deafness. Quality of products from different manufacturers may be varied. Since the current standard could not control the quality of products in a comprehensive and effective way, this study aimed at establishing a practical and convenient approach for holistic quality control of the preparation. This study included both qualitative and quantitative works to get information on the overall composition and main components, respectively. As a result, HPLC fingerprint (UV 240 nm) similarities of all fifty samples were in the range of 0.65∼0.99. Results indicated that there was a difference among products from different manufacturers. Additionally, ten characteristic peaks of the fingerprint were tentatively identified by LC-MS. Further chemometrics analysis was utilized to evaluate the products from different manufacturers. At the same time, the HPLC (UV 285 nm) multicomponent quantification result showed that contents of gentiopicrin, baicalin, baicalein, and wogonin were in the range of 0.61–5.40, 1.96–5.33, 0.10–3.40, and 0.046–1.16 mg·g^−1^, respectively. Data analysis verified the main different component of baicalein from the fingerprint statistical analysis. It is worth mentioning that the qualitative fingerprint and quantitative multicomponent determination were simultaneously accomplished by HPLC-DAD with dual channels. The study provided sound basis for improving quality control standards. This study also provided practical strategy for overall quality control of traditional Chinese medicines.

## 1. Introduction

Long dan Xie gan pill is prepared from ten species of crude drugs including *Gentianae radix* et rhizoma, *Scutellariae radix*, and *Akebiae caulis* (Mutong) in Chinese Pharmacopoeia (2015 Edition, Volume I) [1]. It is widely used for the treatment of diseases, including hepatocolic hygropyrexia, dizziness, tinnitus and deafness, hypochondriac pain [1]. The preparation has attracted widespread attention, since it caused aristolochic acid nephropathy (AAN) [2, 3]. At that time, the prescription collected the crude drug of Caulis aristolochiae manshuriensis (Guanmutong) instead of *Akebiae caulis* [4–6]. Because of the serious adverse effect of aristolochic acids, the medicinal standard of Caulis aristolochiae manshuriensis was abolished and replaced by *Akebiae caulis* without containing such toxic constituents since 2003.

As one commonly used Chinese patent medicine (CPM) with a long history, Long dan Xie gan pill has about 200 manufacturers. Therefore, the quality of products from different manufacturers may be varied. Since quality is directly related to drug safety and efficacy, it is very important to evaluate the holistic quality of the products. Researchers have been working on the essential quality control and evaluation methods for years [2]. For most CPMs, the effective components are not clear and the consistency of product quality is a key indicator of quality product evaluation. It is worth mentioning that fingerprint is an internationally recognized effective method because it could reflect the overall quality information [2–10]. However, fingerprint is usually used for qualitative consistency evaluation. And the quantification could be achieved by applying multicomponent determination [10–13]. In recent years, more and more chromatographic and spectroscopic methods including LC, LC-MS^n^, and quantitative nuclear magnetic resonance (QNMR) are applied for the aforementioned qualitative and quantitative work [14–21]. Among these methods, high-performance liquid chromatography (HPLC) is still the main method deployed in quality control of traditional Chinese medicines (TCMs) because of its advantages, including good repeatability, wide application, and high efficiency.

In this study, the qualitative and quantitative consistency information of Long dan Xie gan pill samples was achieved at the same time by a combination of fingerprint with multicomponent quantification by HPLC-diode array detector (DAD) with dual channels (UV 240 nm and 285 nm). Additionally, further deep mining of the data by chemometrics analysis helped to evaluate the differences of products in a more comprehensive and effective way. The results indicated that the established method could comprehensively analyze the product quality. This strategy could provide a practical approach for the holistic quality control of TCM.

## 2. Materials and Methods

### 2.1. Chemicals and Reagents

Gentiopicrin (97.6%, batch no. 110770-201716), baicalin (93.5%, batch no. 110715-201720), baicalein (98.5%, batch no. 111595-201607), and wogonin (100%, batch no. 111514-201605) were from the National Institutes for Food and Drug Control, Beijing, China. Methanol (analytical reagent) was from National Drug Chemical Reagents Co. Ltd. Acetonitrile (chromatographic pure) and formic acid (mass spectrometry reagent) were from Thermo Fischer Scientific. The water was of ultrahigh purity.

### 2.2. Materials

Fifty batches of Long dan Xie gan pill samples were from 8 manufacturers (A∼H). All the samples involve two dosage forms including water-bindered pills (WBP) and big candied pills (BCP). The detailed information is listed as follows (Table 1).

### 2.3. Instrumentations

HPLC analysis was performed on a Waters 2690 HPLC instrument (Waters, Milford, USA), equipped with a DAD, an autosampler and a column heater. METTLER XS105 electronic analytical balance (Mettler-toledo, Zurich, Switzerland), Milli-Q water purification system (Milli-pore, Burlington, USA), and KQ-300DA numerical control ultrasound cleaning instrument (Kunshan Ultrasonic Instruments Co. Ltd., Kunshan, China) were used. Chemometrics analysis was achieved by ChemPattern software (Chenmind Technologies Co., Ltd., Beijing, China).

### 2.4. Preparation of Standard Solutions

Standard stock solutions of baicalin (0.1 mg·mL^−1^) were prepared by dissolving suitable amounts of reference substance in methanol for fingerprint establishment.

Standard stock mixed solution of gentiopicrin (0.4972 mg·mL^−1^), baicalin (0.5268 mg·mL^−1^), baicalein (0.4688 mg·mL^−1^), and wogonin (0.5080 mg·mL^−1^) was prepared by dissolving suitable amounts of each reference substance in methanol for multicomponents assay.

### 2.5. Preparation of Sample Solutions

For Long dan Xie gan pill (WBP) (6 g per small bag), 5 bags were mixed and pulverized to powder. For Long dan Xie gan pill (BCP) (6 g per pill), 5 pills were cut into small pieces. Then, 2 g were weighed accurately and put into a 50 mL plug conical bottle. Twenty-five mL methanol (for WBP samples) and 25 mL 80% methanol-water solution (for BCP samples) were added precisely and weighed, respectively. After extracting by ultrasonication (power: 300 W; frequency: 40 kHz) for 30 min, the extract was cooled down and then made up for lost weight by adding methanol (for WBP samples) or 80% methanol (for BCP samples). The continuous filtrate was taken and then filtered by 0.22 *μ*m microporous filter membrane.

### 2.6. HPLC-DAD Chromatographic Condition

**Figure 1:**
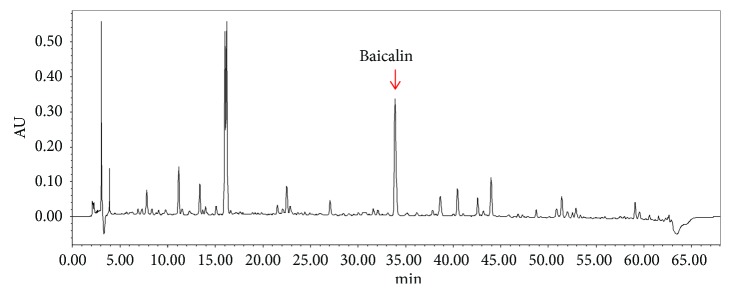
HPLC (UV 240 nm) chromatogram of the typical sample.

**Figure 2:**
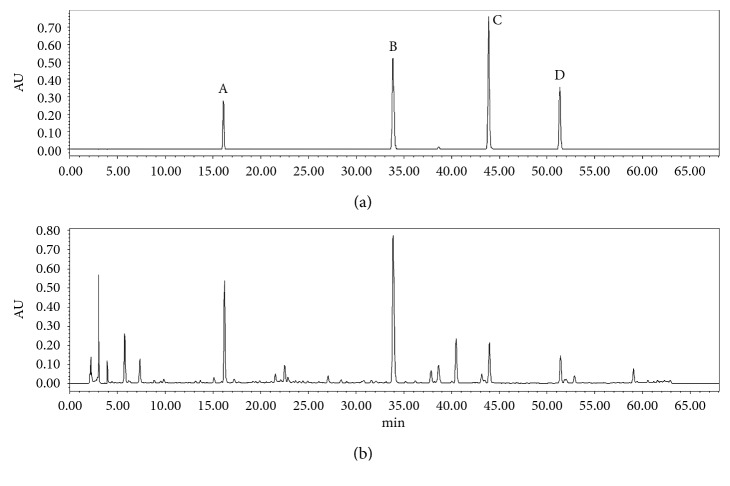
HPLC (UV 285 nm) chromatograms of mixed standard solution (a) (A, Gentiopicrin; B, Baicalin; C, Baicalein; and D, Wogonin) and typical sample (b).

Column: Phenomenex Gemini C18 (4.60 × 250 mm, 5 *μ*m); mobile phase: gradient elution with acetonitrile- (A-)0.1% formic acid-water solution (B) (0–5 min, 7%A–10%A; 5–11 min, 10%A–15%A; 11–15 min, 15%A–20%A; 15–32 min, 20%A–30%A; 32–54 min, 30%A–55%A; 54–60 min, 55%A–80%A; and 60–68 min, 7%A); flow rate: 1.0 mL·min^−1^; column temperature: 30°C; injection volume: 10 *µ*L; detection wavelength: UV 240 nm for fingerprint and UV 285 nm for multicomponent determination. The typical chromatograms were shown as Figures 1 and 2, respectively.

### 2.7. Mass Spectrometry Condition

MS analysis was performed on an Agilent1260-6410B LC-MS couplet system equipped with Agilent Mass Hunter ChemStation (Agilent, Santa Clara, USA). The mass spectrometry settings were as follows: split ratio = 1 : 9; desolvation temperature: 350°C; desolvation air flow N_2_: 540 L·h^−1^; nebulizer pressure: 30 psi; and capillary: 4000 V. Both positive and negative modes were performed with a scan range of *m*/*z* 50–1200.

## 3. Results and Discussion

### 3.1. Results of Fingerprint

For fingerprint study, all the samples were prepared and analyzed according to conditions under 2.5 and 2.6. Baicalin (*t*_R_ = 34.02 min) was taken as the reference peak. Relative retention times (RRTs) and relative peak areas (RPAs) of the characteristic peaks were calculated for method validation.

#### 3.1.1. Instrument Precision

The same sample solution (no. 21) was injected for six consecutive times. The result showed that the RSDs of RRTs and RPAs were in the range of 0.073%–2.0% and 0.26–1.74%, respectively. It showed that the precision of the instrument was good.

#### 3.1.2. Repeatability

The same batch sample (no. 21) was taken and prepared for six independent sample solutions for analysis. The result showed that the RSDs of RRT and RPA were in the range of 0.010%–0.44% and 0.51–2.24%, respectively. It indicated that method repeatability was good.

#### 3.1.3. Stability

The same sample solution (no. 21) was injected at 0, 4, 8, 12, 16, 20, and 24 h at room temperature. The result showed that the RSDs of RRT and RPA were in the range of 0.039%–0.81% and 0.71–4.58%, respectively. It demonstrated the sample solution was stable within 24 h.

#### 3.1.4. Establishment of Fingerprint

**Figure 3:**
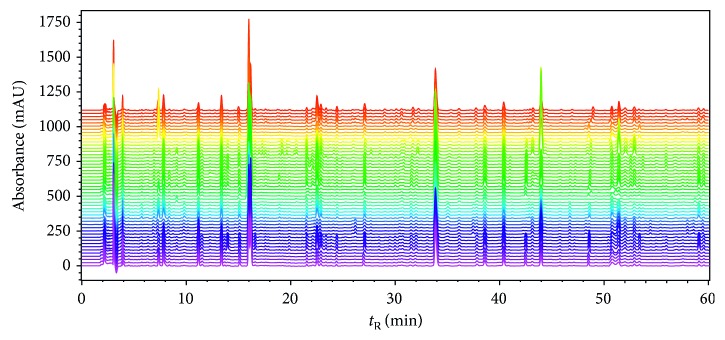
HPLC chromatograms (UV240 nm) of Long dan Xie gan pill samples.

**Figure 4:**
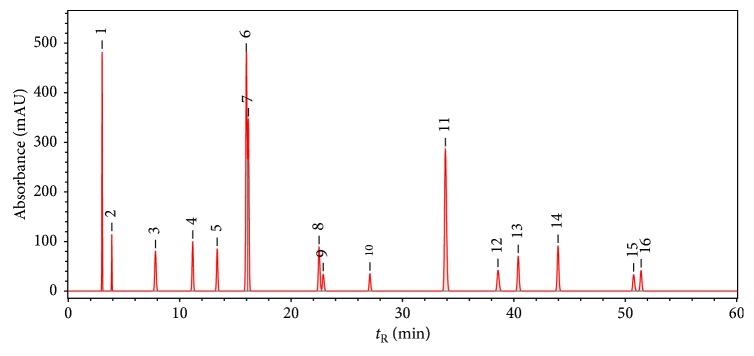
HPLC (UV240 nm) common mode of Long dan Xie gan pill samples.

After fifty batches of the sample solutions were analyzed, their chromatograms (UV 240 nm) were recorded (Figure 3) and imported to ChemPattern software. All variables were used as the common peak screening condition. And Gauss curve simulation method was applied to generate the common mode with 16 characteristic peaks (Figure 4). All sample chromatograms were analyzed by comparison with the common mode.

#### 3.1.5. Identification and Attribution of Characteristic Peaks

The sample solution was analyzed according to the conditions under Sections 2.6 and 2.7. By combination with the chromatographic behavior of the components, ten characteristic peaks were identified by comparing with reference standards. Also the main origins of the peaks were attributed (Table 2), and they were mainly from five species of crude drugs in the prescription.

#### 3.1.6. Statistical Analysis

**Figure 5:**
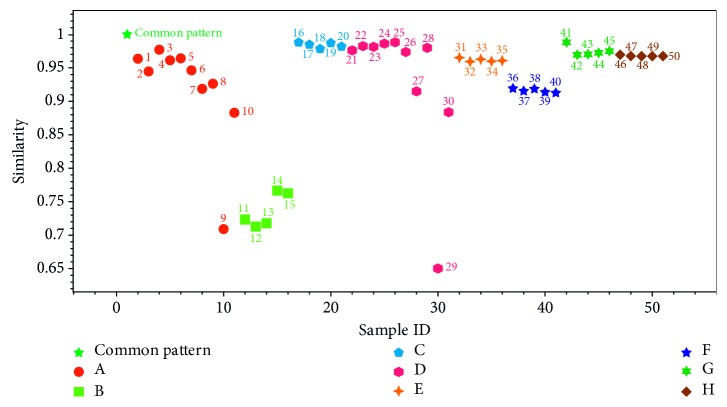
Similarity analysis results of Long dan Xie gan pill samples.

*(1) Similarity Analysis*. The above HPLC (UV 240 nm) fingerprint common mode was taken as a reference. During the analysis, the included angle cosine method was used to calculate the similarity of each sample (Figure 5). Finally, the similarities of all samples were in the range of 0.65∼0.99. Among them, similarities of seven batches of samples were lower than 0.8, including all samples from enterprise B. The result indicated that there was a certain difference among the overall product quality of these samples from others. Also it was clear that the uniformity of most BCP samples (no. 6∼10 and 26∼30) was not good as the WBP ones.

**Figure 6:**
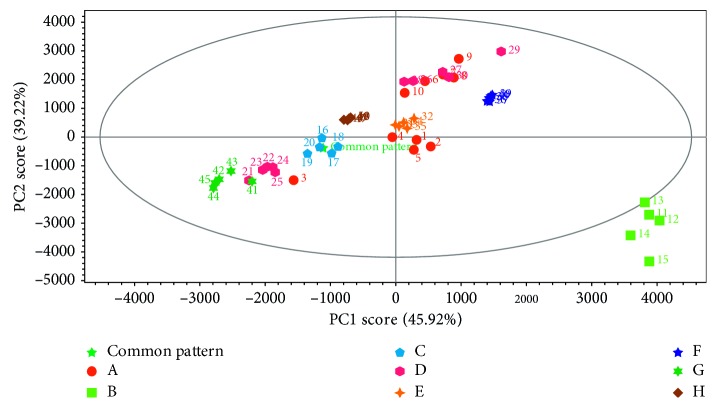
PCA score scatter plot of Long dan Xie gan pill samples.

**Figure 7:**
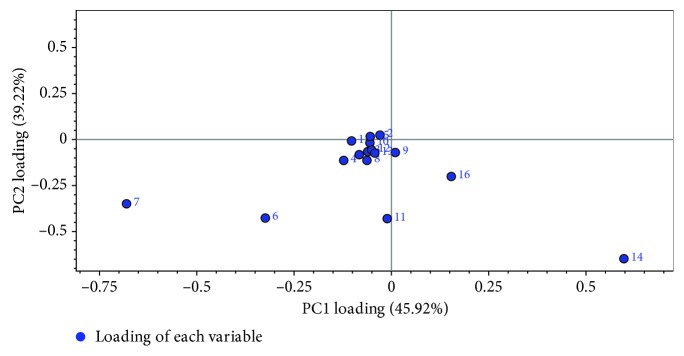
PCA loading scatter plot of Long dan Xie gan pill samples (1–16 represents the number of chromatographic peaks in the common mode).

*(2) Principle Component Analysis*. Principle component analysis (PCA) was carried out after standardization of all sample data (Figures 6 and 7). The contribution rates of the first and second principle component (PC1) were 45.92% and 39.22%, respectively. And the total contribution rate of 85.14% showed that it could reflect the differences between samples in a more comprehensive way. PCA scatter plot (Figure 6) displayed that samples from each enterprise basically could be grouped into a class. It showed that samples from enterprise B deviated far away from others. The principle component load diagram (Figure 7) gave the proportion of each chromatographic peak in the principal component. And the greater the distance from *X* = 0 longitudinal axis, the greater the contribution to PC1, such as gentiopicrin and baicalein. Likewise, the greater the distance from *Y* = 0 transverse axis, the greater the contribution to PC2, such as baicalein and geniposide. The result displayed that samples from enterprise B separated with others along both PC1 and PC2. Therefore, the major contributions to PC1 and PC2 were their main differential components from others. Because of the location at the position of PC1 > 0 and PC2 < 0, contents of such compounds were in positive correlation with PC1 and in negative correlation with PC2. Therefore, the contents of baicalein and wogonin were higher in these samples. Along the PC1, the concentration of baicalein distinguished the samples of enterprise B, whose values were higher than those presented for the others (Figure 6). On the contrary, along with the PC2, the levels of geniposide and gentiopicrin showed that there was a tendency for the separation of the products of enterprises D and G from others. One sample of enterprise A was grouped with samples of enterprises D and G, which presented higher levels for these compounds.

**Figure 8:**
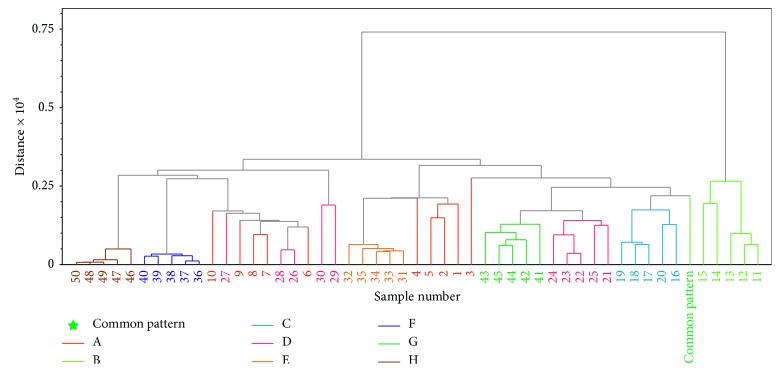
Dendrograms of hierarchical cluster analysis of Long dan Xie gan pill samples.

*(3) Cluster Analysis*. Hierarchical cluster analysis (HCA) is a conventional cluster analysis method. It is a detection tool that clearly reveals the natural grouping of data. The block distance was selected for distance calculation and HCA (Figure 8) was performed by error square sum method. Similar to the result of PCA, except that some samples from manufacturer D are not distinguished from those of A, other samples from different enterprises could basically distinguish. Additionally, samples from B were relatively most far away from others. It indicated there existed some differences of these samples.

### 3.2. Results of Multicomponent Quantification

#### 3.2.1. Linearity, LOD, and LOQ

Working standard solutions containing gentiopicrin, baicalin, baicalein, and wogonin were prepared by diluting the stock mixed solution with methanol to a series of proper concentrations. Then, they were injected and analyzed. The results of regression equations, linearity, determination coefficient, and limits of detection and quantification of the method are presented in Table 3. The linear range varied from 3.25 to 492.56 *μ*g mL^−1^, in accordance with the analyte. All analytes presented a determination coefficient (*R*^2^) of the 0.9999, which allows the method to be considered linear. The limits of detection (LOD) and quantification (LOQ) were calculated according to guidelines for validation of analytical methods for pharmaceutical quality standards [22].

#### 3.2.2. Instrument Precision

The same sample solution (no. 21) was injected for six consecutive times and analyzed. The RSDs of peak areas for gentiopicrin, baicalin, baicalein, and wogonin were 0.63%, 0.29%, 0.41%, and 0.15%, respectively. It indicated that the precision of the instrument was in accordance with the requirement in guidelines for validation of analytical methods for pharmaceutical quality standards [22].

#### 3.2.3. Repeatability

The same batch of sample (no. 21) was taken and prepared for six independent sample solutions. Then, they were analyzed according to conditions under 2.6. The average contents of gentiopicrin, baicalin, baicalein, and wogonin were 4.77, 3.84, 0.62, and 0.40 mg·g^−1^, respectively. And the RSDs were 0.49%, 1.11%, 0.40%, and 0.56%, respectively. It indicated that method repeatability was in accordance with the requirement in guidelines for validation of analytical methods for pharmaceutical quality standards [22].

#### 3.2.4. Stability

The same sample solution (no. 21) was injected at 0, 4, 8, 12, 18, and 24 h at room temperature. The RSDs of contents for gentiopicrin, baicalin, baicalein, and wogonin were 1.88%, 1.54%, 1.70%, and 3.22%, respectively. It indicated that the sample solution was stable within 24 h.

#### 3.2.5. Recovery

The recovery experiment was performed by adding a known amount of individual reference standards into a certain amount of sample (no. 21).

Six separate samples of 1 g (contents of gentiopicrin, baicalin, baicalein, and wogonin were 4.77, 3.84, 0.62, and 0.40 mg·g^−1^, respectively) were weighed accurately. And 25 mL of mixed reference standard solution (concentrations of gentiopicrin, baicalin, baicalein, and wogonin were 0.1861, 0.1505, 0.04856, and 0.0300 mg·mL^−1^, respectively) was added separately and prepared. The results (Table 4) showed that the average recoveries of four components ranged from 97.71% to 100.59% with RSDs in the range of 0.72%–1.29%, which indicated that the method was accurate.

#### 3.2.6. Sample Analysis

**Figure 9:**
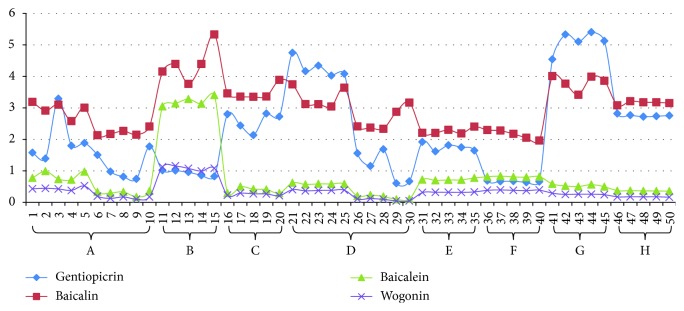
The scatter diagram of four components' contents in Long dan Xie gan pill samples.

Fifty batches of sample solutions were prepared and analyzed. The results (Table 5) displayed that the contents of gentiopicrin, baicalin, baicalein, and wogonin were in the range of 0.61–5.40, 1.96–5.33, 0.10–3.40, and 0.046–1.16 mg·g^−1^, respectively. It was easily to find the differences among samples from different enterprises by the scatter diagram (Figure 9). It showed that the general content trends of baicalin, baicalein, and wogonin were basically similar. Among them, the contents of baicalein and wogonin in samples from B were apparently higher than others; especially, the content of baicalein was much higher. The determination result was in accordance with the abovementioned PCA analysis result.

### 3.3. Optimization of Experimental Conditions

#### 3.3.1. Investigation of Extraction Methods

The extraction method was optimized in order to make the fingerprint reflect the chemical composition information as much as possible. For both dosage forms of samples, different extraction solvent (80% methanol and 50% methanol-water), and extraction mode and time (ultrasonic extraction for 30 min, 45 min, and 60 min) were investigated. The result showed that extraction time had little effect on both dosage forms. For WBP samples, the chromatogram could reflect rich chemical information with good separation of peaks with methanol extraction for 30 min. While for BCP samples, the dissolution was good by extraction with 80% methanol-water for 30 min.

#### 3.3.2. Study on the Chromatographic Condition

During the study, different mobile phase systems including methanol-water, acetonitrile-water, methanol-0.1% formic acid, and acetonitrile-0.1% formic acid were investigated. Also different chromatographic, columns including Waters Symmetry Shield™ RP 18, Agilent Zorbax SB-C18, and Phenomenex Gemini C18 were experimented. Additionally, both DAD and DAD-ELSD detection were analyzed. As a result, the chromatogram was good on the Phenomenex Gemini C18 column with acetonitrile-0.1% formic acid as the mobile phase under DAD detection. Finally, UV 240 nm was selected as detection wavelength for fingerprint establishment, since it could reflect much chemical information. Meanwhile, UV 285 nm was determined as detection wavelength for simultaneous determination of main compounds due to the good separation.

## 4. Conclusions

Quality control is the key issue in modernization and internationalization of TCM. Qualitative fingerprint and quantitative multicomponent determination have been demonstrated as the comprehensive and effective way to accomplish the holistic quality analysis. In this study, both qualitative and quantitative works to get the overall composition and main components information were accomplished simultaneously by HPLC with dual-channel detection. Moreover, further deep mining of the data by chemometrics analysis helped to evaluate the quality of the preparation from different manufacturers. The result indicated that this approach is a powerful tool for quality control of TCM.

## Figures and Tables

**Table 1:** Sample information.

No.	Manufacturers	Batch number	Dosage
1	A	B16099	WBP
2		B17001	
3		B17045	
4		B16033	
5		B16040	
6		A17057	BCP
7		A16065	
8		A16119	
9		A17004	
10		A17104	

11	B	20170502	WBP
12		20170504	
13		20170503	
14		171002	
15		171003	

16	C	1704058	WBP
17		1801024	
18		1801027	
19		1801026	
20		1711016	

21	D	20180336	WBP
22		20180335	
23		20180334	
24		20180452	
25		20180451	
26		20170621	BCP
27		20180419	
28		20170620	
29		20170504	
30		20170308	

31	E	180203	WBP
32		180201	
33		180204	
34		180205	
35		180202	

36	F	171102	WBP
37		171002	
38		170902	
39		171101	
40		171001	

41	G	1801001	WBP
42		1802005	
43		1801003	
44		1801004	
45		1802006	

46	H	180102	BCP
47		180101	
48		171001	
49		171002	
50		160501	

**Table 2:** Identification of characteristic peaks of Long dan Xie gan pill.

Peak no.	Compounds	*t*_R_ (min)	Molecular formula	Molecular weight	Quasimolecular ions	Origin
3	Geniposidic acid	7.83	C_16_H_22_O_10_	374.34	372.9 [M-H]^−^	*Gardeniae fructus*
6	Geniposide	15.07	C_17_H_24_O_10_	388.37	433.0 [M+HCOO]^−^	*Gardeniae fructus*
7	Gentiopicrin	16.18	C_16_H_20_O_9_	356.32	400.9 [M+HCOO]^−^	*Gentianae radix* et rhizoma
8	Verbascoside	22.86	C_29_H_36_O_15_	624.59	623.0 [M-H]^−^	Plantaginis semen/rehmanniae radix
10	Baicalin	33.88	C_21_H_18_O_11_	446.36	445.0 [M-H]^−^	*Scutellariae radix*
11	Isomer of wogonoside	38.62	C_22_H_20_O_11_	460.39	459.0 [M-H]^−^	*Scutellariae radix*
12	Wogonoside	40.44	C_22_H_20_O_11_	460.39	459.0 [M-H]^−^	*Scutellariae radix*
14	Baicalein	43.82	C_15_H_10_O_5_	270.24	268.9 [M-H]^−^	*Scutellariae radix*
15	Isomer of wogonin	51.40	C_16_H_12_O_5_	284.26	282.9 [M-H]^−^	*Scutellariae radix*
16	Wogonin	51.99	C_16_H_12_O_5_	284.26	282.9 [M-H]^−^	*Scutellariae radix*

**Table 3:** Regression equations, linearity, determination coefficient, and limits of detection and quantification of the method.

Components	Regression equations	Linear range (*μ*g·mL^−1^)	*R* ^2^	LOD (ng)	LOQ (ng)
Gentiopicrin	*y* = 11524 *x* + 12562	7.76∼485.27	0.9999	0.837	2.790
Baicalin	*y* = 32266 *x* + 4256.2	7.88∼492.56	0.9999	0.431	1.077
Baicalein	*y* = 43732 *x* − 23130	7.39∼461.77	0.9999	0.439	1.025
Wogonin	*y* = 47996 *x* + 23628	3.25∼203.20	0.9999	0.190	0.474

**Table 4:** Recovery results of four components in Long dan Xie gan pill samples.

Components	No.	Sampling amount (g)	Sample content (mg)	Added amount (mg)	Detected amount (mg)	Recovery (%)	Average recovery (%)
Gentiopicrin	1	1.0005	4.772	4.653	9.371	98.83	100.59% (RSD 1.22%)
	2	1.0017	4.778	4.653	9.432	100.02	
	3	1.0006	4.773	4.653	9.431	100.11	
	4	1.0047	4.792	4.653	9.473	100.60	
	5	1.0030	4.784	4.653	9.528	101.96	
	6	1.0026	4.782	4.653	9.529	102.02	

Baicalin	1	1.0005	3.842	3.762	7.478	96.65	97.96% (RSD 0.87%)
	2	1.0017	3.847	3.762	7.537	98.09	
	3	1.0006	3.842	3.762	7.503	97.32	
	4	1.0047	3.858	3.762	7.583	99.02	
	5	1.0030	3.852	3.762	7.543	98.11	
	6	1.0026	3.850	3.762	7.554	98.56	

Baicalein	1	1.0005	0.620	1.214	1.807	97.78	97.71% (RSD 0.72%)
	2	1.0017	0.621	1.214	1.802	97.28	
	3	1.0006	0.620	1.214	1.793	96.62	
	4	1.0047	0.623	1.214	1.809	97.69	
	5	1.0030	0.622	1.214	1.816	98.35	
	6	1.0026	0.622	1.214	1.818	98.52	

Wogonin	1	1.0005	0.400	0.750	1.141	98.80	99.98% (RSD 1.29%)
	2	1.0017	0.401	0.750	1.146	99.33	
	3	1.0006	0.400	0.750	1.141	98.80	
	4	1.0047	0.402	0.750	1.153	100.13	
	5	1.0030	0.401	0.750	1.156	100.67	
	6	1.0026	0.401	0.750	1.167	102.13	

**Table 5:** Contents of four components in Long dan Xie gan pills (mg·g^−1^).

No.	Manufacturers (dosage)	Gentiopicrin	Baicalin	Baicalein	Wogonin
1	A (WBP)	1.579	3.191	0.778	0.433
2		1.391	2.912	0.992	0.449
3		3.295	3.102	0.741	0.429
4		1.801	2.582	0.718	0.381
5		1.884	3.006	0.976	0.526

6	A (BCP)	1.510	2.133	0.330	0.198
7		0.979	2.173	0.298	0.130
8		0.816	2.269	0.337	0.176
9		0.746	2.148	0.176	0.103
10		1.777	2.409	0.362	0.176

11	B (WBP)	1.019	4.153	3.049	1.129
12		1.013	4.394	3.141	1.158
13		0.963	3.763	3.280	1.080
14		0.860	4.396	3.133	0.994
15		0.826	5.330	3.405	1.084

16	C (WBP)	2.801	3.460	0.295	0.232
17		2.442	3.359	0.502	0.300
18		2.138	3.353	0.423	0.274
19		2.822	3.363	0.411	0.270
20		2.724	3.889	0.294	0.216

21	D (WBP)	4.753	3.747	0.622	0.406
22		4.166	3.123	0.570	0.367
23		4.344	3.118	0.590	0.381
24		4.028	3.047	0.585	0.385
25		4.084	3.642	0.589	0.390

26	D (BCP)	1.562	2.412	0.203	0.110
27		1.157	2.371	0.235	0.125
28		1.688	2.337	0.189	0.102
29		0.606	2.879	0.100	0.046
30		0.670	3.170	0.111	0.052

31	E (WBP)	1.922	2.210	0.722	0.314
32		1.620	2.205	0.698	0.324
33		1.815	2.304	0.715	0.323
34		1.749	2.188	0.715	0.320
35		1.650	2.407	0.779	0.333

36	F (WBP)	0.690	2.297	0.817	0.387
37		0.669	2.279	0.837	0.397
38		0.675	2.175	0.811	0.385
39		0.640	2.051	0.800	0.374
40		0.660	1.963	0.821	0.394

41	G (WBP)	4.544	4.012	0.587	0.291
42		5.331	3.771	0.524	0.256
43		5.103	3.419	0.505	0.263
44		5.402	3.990	0.567	0.261
45		5.128	3.862	0.501	0.249

46	H (BCP)	2.822	3.082	0.372	0.176
47		2.768	3.214	0.376	0.181
48		2.721	3.177	0.367	0.178
49		2.736	3.177	0.365	0.180
50		2.758	3.153	0.359	0.168

## Data Availability

The data used to support the findings of this study are available from the corresponding author upon request.
